# An update on gastrointestinal endoscopy-associated infections and their contributing factors

**DOI:** 10.1186/s12941-018-0289-2

**Published:** 2018-10-10

**Authors:** Charles Eugenio McCafferty, Marra Jai Aghajani, David Abi-Hanna, Iain Bruce Gosbell, Slade Owen Jensen

**Affiliations:** 1 Ingham Institute for Applied Medical Research, 1 Campbell Street, Sydney, NSW 2560 Australia; 2 Western Sydney University, School of Medicine, Sydney, 2560 Australia; 3 Department of Gastroenterology and Hepatology, Liverpool Hospital, Sydney, 2170 Australia; 4 School of Medicine, University of New South Wales, Sydney, 2033 Australia

## Abstract

**Introduction:**

During clinical use, gastrointestinal endoscopes are grossly contaminated with patient’s native flora. These endoscopes undergo reprocessing to prevent infectious transmission upon future use. Endoscopy-associated infections and outbreaks have been reported, with a recent focus on the transmission of multi-drug resistant organisms. This review aims to provide an update on endoscopy-associated infections, and the factors contributing to their occurrence.

**Methods:**

PubMed, ScienceDirect, and CINAHL were searched for articles describing gastrointestinal endoscopy-associated infections and outbreaks published from 2008 to 2018. Factors contributing to their occurrence, and the outcomes of each outbreak were also examined.

**Results:**

This review found 18 articles, 16 of which described duodenoscope-associated infections, and the remaining two described colonoscope- and gastroscope-associated infection respectively. Outbreaks were reported from the United States, France, China, Germany, the Netherlands and the United Kingdom. The causative organisms reported were *Klebsiella pneumoniae*, *Pseudomonas aeruginosa*, *Escherichia coli* and *Salmonella enteritidis*.

**Conclusions:**

A number of factors, including lapses in reprocessing, biofilm formation, endoscope design issues and endoscope damage, contribute to gastrointestinal endoscopy associated infection. Methods of improving endoscope reprocessing, screening for contamination and evaluating endoscope damage may be vital to preventing future infections and outbreaks.

## Background

During clinical use, endoscopes are contaminated with patients’ native flora [1]. These infectious agents must be removed to prevent cross-infection during subsequent use [2–4]. Endoscopes usually undergo reprocessing rather than sterilization [4]. Endoscope reprocessing is performed in Australia according to the guidelines provided by the Gastroenterological Nurses College of Australia and Gastroenterological Society of Australia (GENCA/GESA) [5]. Reprocessing typically consists of 8 steps: precleaning, leak testing, manual cleaning, rinsing after cleaning, visual inspection, high-level disinfection, rinsing after high-level disinfection, and drying [5].

Endoscopes cannot be autoclaved because they are made of heat-sensitive materials [6]. There are low-temperature alternatives, including ethylene oxide (EtO) gas sterilisation, which have been adopted in response to culture-positive endoscopes and endoscopy-associated outbreaks [7, 8]. EtO gas sterilization is more expensive than standard reprocessing, requires more time, poses a risk to the health of associated staff, and has been suggested to shorten an endoscopes lifespan [7, 9, 10].

Endoscope reprocessing has been associated with a variable failure rate, one study reporting gastroscope and colonoscope reprocessing failure rates of 1.8% and 1.9% respectively [3]. There are many reasons for persistent contamination including failures in reprocessing, acquired and inherent endoscope defects, inappropriate or defective cleaning supplies, and biofilm formation [11]. Persistent endoscope contamination can facilitate endoscopy-associated infections and outbreaks [12].

The risk of infectious transmission was previously estimated to be 1 in 1.8 million endoscopic procedures [13]. This figure now appears to be a significant underestimation for many reasons, including a lack of detailed surveillance for infections following endoscopy, underreporting, and a lack of recognition of acknowledged transmissions [14]. The significance of these transmission events has increased with the emergence of multidrug-resistant organisms and their involvement in endoscopy-associated outbreaks [15, 16].

To prevent endoscopy-associated infection, it is recommended that endoscopy units perform surveillance cultures [5, 17]. In Australia, duodenoscopes, bronchoscopes, linear EUS (endoscopic ultrasound) endoscopes and automatic endoscope/flexible endoscope reprocessor (AERs/AFERs) undergo monthly testing, and all other endoscopes, including gastroscopes and colonoscopes, are tested quarterly [17]. Surveillance cultures are retrospective and act to limit rather than prevent patient exposure. As such, endoscopy-associated infections can still occur despite reprocessing and surveillance cultures. This review aimed to provide an update on published gastrointestinal endoscopy-associated infections and outbreaks, and explore the factors contributing to their occurrence.

## Methods

The primary investigator created the search strategy, which was reviewed by each of the authors. PubMed, ScienceDirect and CINAHL were searched for full-text articles published in English within the last 10 years, and an additional filter, “human” was applied to the PubMed search.

Searches were performed with the following keywords: “endoscop*”, “gastroscop*”, “colonoscop*”, “duodenoscop*”, “endoscopic retrograde cholangiopancreatography”, “gastrointestinal endoscop*”, “clean*”, “reprocess*”, “steril*”, “high?level disinfection”, “infect*”, “contaminat*”, “outbreak*”, and “vector*”. Across all three of the databases, a total of 1500 articles were found. Article titles were screened before two reviewers independently screened abstracts of the selected articles. Articles discussing an outbreak or transmission event related to a gastrointestinal endoscope were selected. Full-texts were then downloaded (333) and reviewed.

**Fig. 1:**
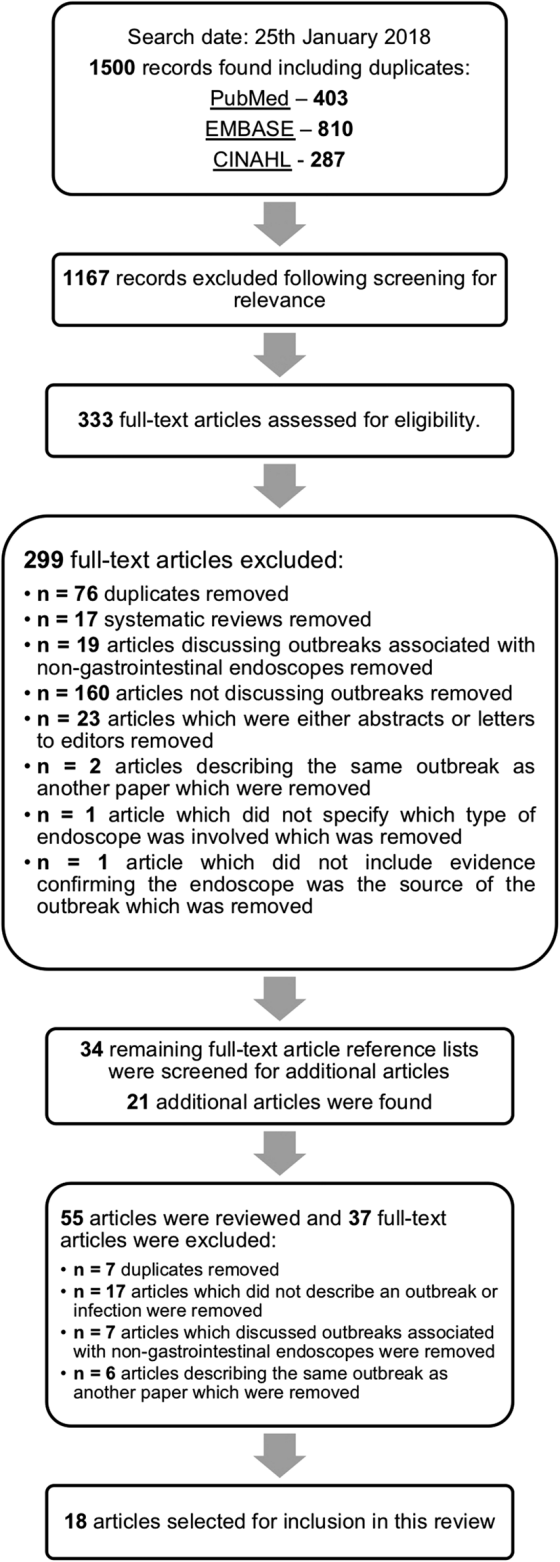
Search strategy and study selection flow chart. This is a flow chart illustrating which databases were searched and the number of articles found from each database. This flow chart also documents the screening process and reasons for the exclusion of articles from the review, as well as the number of articles removed at each screening step

Articles were excluded if they did not describe an endoscopy-associated infection(s) or outbreak(s) or described an infection transmission event involving a non-gastrointestinal endoscope. Articles were included if they described case(s) of endoscopy-associated infection(s) or outbreak(s), were written in English, and were published within the last 10 years. The search strategy and study selection process are summarised in Fig. 1.

## Results

Of the 18 articles reviewed, sixteen described outbreaks associated with duodenoscope use, and two described outbreaks associated with gastroscope and colonoscope use respectively. The causative microorganisms reported were *Klebsiella pneumoniae*, *Pseudomonas aeruginosa*, *Escherichia coli*, and *Salmonella enteritidis*.

### Outbreaks related to reprocessing

Nine articles reported issues with endoscope reprocessing [18–26]. Issues with drying were reported in five of these studies. Poor adherence to cleaning guidelines was reported in seven, and one article described reprocessing prior to the introduction of national standards [23].

Naas et al. [20] and Carbonne et al. [18] describe a multi-hospital outbreak of carbapenemase-producing *Klebsiella* pneumoniae associated with a contaminated duodenoscope [15]. Seventeen patients were exposed to the duodenoscope, ten were screened and six were found to be colonised with *K. pneumoniae*. A review of the reprocessing method revealed issues with drying. Following this, drying was prioritised and the frequency of duodenoscope microbiological surveillance was increased to a monthly basis.

Aumeran et al. [22] also report on a duodenoscope-associated outbreak with extended-spectrum beta lactamase-(ESBL) producing *K. pneumoniae*. Similarly, a reprocessing audit revealed endoscopes were not fully dried before storage and were brushed before being flushed with a detergent solution. The endoscope remained culture negative until a Tween-based solution was used. The duodenoscopes were inspected, and reprocessing adherence was mandated. An additional day was allocated for procedures to decrease the number of procedures per day.

Bajolet et al. [21] describe a gastroscope-associated outbreak in four patients with an ESBL-producing *P. aeruginosa*. A reprocessing audit revealed that the time dedicated to brushing/flushing of endoscope channels was less than 10 min, suction cylinders were not sterilised, a single diameter channel cleaning brush was used for all gastrointestinal endoscopes, and the drying process was inadequate. Following this, compliance with the recommended manual cleaning time, sterilisation of suction cylinders and quarterly microbiological surveillance testing was enforced.

Alrabaa et al. [19] and Sanderson et al. [24] report the infection of seven patients with carbapenemase-producing *K. pneumoniae*. A reprocessing audit found deviation during duodenoscope elevator channel cleaning, such that debris remained underneath the elevator piece. Culturing of this section revealed *E. coli*. Of 51 exposed patients, 46 underwent screening and three patients were colonised with carbapenemase-producing organisms.

Reiner et al. [25] describe an outbreak of *P. aeruginosa* bacteraemia and sepsis in three patients following endoscopic retrograde cholangiopancreatography (ERCP) with the same duodenoscope. Cultures from 6 of the 12 endoscopes, including the implicated endoscope, grew *P. aeruginosa*. Fluid samples from bottles containing enzymatic solution for precleaning revealed the presence of *P. aeruginosa*. These bottles were all topped off by a larger bottle, which tested positive for *P. aeruginosa* and *S. maltophila*. The outbreak was terminated after the concentration of solution used was standardized, refillable enzymatic bottles were removed and replaced with single case enzymatic packs, and competencies for reprocessing personnel were repeated.

Robertson et al. [23] postulate that a patient colonised with *Salmonella enteritidis* underwent ERCP and contaminated a loaned duodenoscope which then transmitted the *S. enteritidis* spp. to three other patients. A reprocessing audit revealed issues including reuse of endoscope cleaning brushes, no dedicated sink for hand hygiene, no commissioning data for the endoscope washer disinfection (EWD), the EWD failed to provide vital information such as channel patency and failed a subsequent load dryness test. Following the outbreak, the EWD was removed from service.

Reddick [26] also reported an outbreak involving *S. enteritidis*, with three patients following colonoscopy with the same colonoscope. Pulse-gel field electrophoresis patterns for each isolate were identical, but the implicated colonoscope was culture negative. A reprocessing review found that clean and soiled endoscopes were kept in the same room, and endoscopes were kept in the AER when there was no hanging space available for drying.

### Outbreaks associated with no reprocessing lapses or endoscope defects

Seven studies reported outbreaks with no issues in reprocessing or endoscope defects [7, 27–31]. Other published outbreaks have also occurred despite no apparent lapses in reprocessing [9, 29, 32]. In four articles, implementation of EtO sterilisation limited the outbreak.

Kola et al. [28] report an outbreak of OXA-48 carbapenemase-producing *K. pneumoniae* (CRKP). The authors hypothesise an infected patient contaminated a duodenoscope, which then infected five other patients. A reprocessing audit revealed no deviations, but cultures performed on another endoscope grew enterococci.

Humphries et al. [30] describe an outbreak of multidrug-resistant *Klebsiella pneumoniae* bacteraemia and sepsis following ERCP, of which two of nine infected patients died as a result. A reprocessing review found no deviations, and the endoscopes were culture negative, except for 1–2 colonies of coagulase-negative *Staphylococcus*. In response, implicated scopes were retired, reprocessing was modified and duodenoscopes and linear echoendoscopes underwent EtO gas sterilization off-site. Additional duodenoscopes were purchased, reprocessing staff repeated competencies for both endoscopes and AERs, and reprocessing underwent weekly observation. Following these changes, no further outbreaks or infections were reported.

Qiu et al. [29] report an outbreak involving a duodenoscope contaminated with *P. aeruginosa*. The authors hypothesise a patient contaminated the duodenoscope, which then infected another two patients. The duodenoscope underwent four rounds of reprocessing but remained culture-positive. The endoscope manufacturers then sterilised the endoscope with epoxyethane, dismantled the channels, and examined them for biofilm, which was absent.

Kovaleva et al. [31] describe an outbreak of multidrug-resistant *P. aeruginosa* with 3 patients following ERCP. Following the second case, the duodenoscope was cultured and produced *P. aeruginosa*. Despite multiple rounds of reprocessing, the duodenoscope remained culture-positive. The duodenoscope was then removed from service and underwent EtO gas sterilisation, after which the duodenoscope was culture negative. Four months later, the duodenoscope was culture positive for *P. aeruginosa*. After inspection, structures suggestive of biofilm were identified in undamaged channels of the scope.

Kim et al. [27] report an outbreak of CRKP with two epidemiologically linked culture-negative duodenoscopes. No breaches were identified in the reprocessing method. One hundred and fifteen patients were exposed, 104 patients underwent screening and 15 were infected with CRKP. Biliary stent placement, cholangiocarcinoma and active inpatient status were each found to increase the risk of developing carbapenemase-resistant Enterobacteriaceae (CRE) infection following ERCP. The risk of clinically relevant CRE infection following ERCP was calculated at 7.7%.

Both Smith et al. [9] and Epstein et al. [7] report an outbreak of New Delhi metallo-beta-lactamase 1-(NDM-1) producing *E. coli* associated with contaminated duodenoscopes despite no reprocessing lapses. In response, both units implemented EtO sterilisation of duodenoscopes and no further cases were reported. Epstein et al. [7] report that the associated duodenoscopes were culture-negative.

### An outbreak associated with endoscope defects

Wendorf et al. [33] describe an outbreak of AmpC *E. coli* associated with ERCP. An investigation found 32 cases following exclusion of secondary infection transmission and duplicate cases. A reprocessing audit revealed the unit was reprocessing above the industry standard. However, seven of the eight duodenoscopes inspected by the manufacturer had at least one critical defect; three failed a leak test during the manufacturers assessment but passed at the hospital.

### An outbreak associated with endoscope design

Verfaillie et al. [32] report on an outbreak related to the design of a specific duodenoscope, the Olympus TJF-180 V, which has since been removed from clinical use. The duodenoscope had a fixed distal cap, and a sealed elevator wire channel port; sealed with an O-ring, which may not have sealed the forceps elevator axis sufficiently. Thirty patients were infected with a VIM-2-positive *P. aeruginosa* strain, 22 of which were infected by the duodenoscope. Following removal of the model from clinical use, the number of VIM-2-positive *P. aeruginosa* cases decreased.

Scanning electron microscopy (SEM) of the distal cap and its components revealed a rough surface on the O-ring, as well as sludge behind the glass that covers the light-guide lens, a crack in the fixed cap, a brown layer on the O-ring and brown staining of the frame of the distal tip. There were no breaches in adherence to the reprocessing guidelines.

## Discussion

Eighteen outbreaks associated with gastrointestinal endoscopy were reviewed. The majority were associated with duodenoscopes, the distal tip of which could not be dismantled to facilitate effective cleaning [32] and contains an intricate contamination-prone elevator mechanism [34]. Recently, duodenoscopes with detachable distal caps have been developed to address this issue [35].

Outbreaks were also associated with lapses in endoscope reprocessing. Manual cleaning, which is crucial to preventing biofilm formation [36], was identified as inadequate [19, 21–23] in four of the reviewed articles. In contrast, seven outbreaks occurred with no apparent reprocessing lapses, which could be explained by biofilm contamination [29]. It has been shown that biofilm can persist within an endoscope despite reprocessing [37], and this may contribute to persistent contamination. In one article, structures suggestive of biofilm were found inside the channels of a persistently contaminated duodenoscope [31]. Kovaleva specifically identified *P. aeruginosa* as a biofilm-producer [38], which is interesting as it was the culprit organism in three outbreaks.

The drying process is also crucial to effective endoscope reprocessing [17, 39]. Recently, the use of drying cabinets with pressurised air flow through endoscope channels has been shown to reduce microbiological load [40, 41], and has been implemented in endoscopy units [5] to ensure thorough drying.

Endoscope defects are also a potential explanation, as identified by Wendorf et al. [33]. An article by Ofstead et al. used borescopes to identify damage within endoscope channels, and found previously undetected damage and debris [42]. This damage may facilitate persistent contamination despite adherent reprocessing, and potential biofilm formation.

Furthermore, endoscope reprocessing can have a variable failure rate [3]. A study following the outbreak reported by Wendorf et al. found that despite stringent reprocessing, there was an inherent high-level disinfection failure rate of 2% [43]. Similar rates were identified by Bisset et al. for gastroscopes and colonoscopes (1.8% and 1.9% respectively) [3], and by Higa et al. for endoscopes (1.9%) [44].

The exclusion of outbreaks that were not published in a peer-reviewed journal, and the low number of published outbreaks, limit this review. Most endoscope reprocessing lapses are never reported [11] or associated infections are not recognised [14], and if an outbreak has not been contained, it may not be reported [15]. Gastmeier and Vonberg suggest in their own review of endoscopy-associated infections with *Klebsiella* spp. that it is very likely outbreaks may not be acknowledged because they involve commensal bacteria of the gastrointestinal tract [45]. The review is also limited by the quality of evidence; endoscopy-associated infections are usually reported as case studies or case series.

## Conclusion

In conclusion, methods of improving endoscope reprocessing and screening for endoscope contamination, such as the use of adenosine triphosphate (ATP) measurement [46], may be vital to preventing future endoscopy-associated infections and outbreaks. Furthermore, endoscope reprocessing may be more effective if it is regularly reviewed to ensure adherence, and routine maintenance and inspection is crucial to preventing infection transmission.

## Abbreviations

GENCA/GESA: Gastroenterological Nurses College of Australia and Gastroenterological Society of Australia
EtO: ethylene oxide
EUS: (endoscopic ultrasound) endoscope
AER/AFER: automatic endoscope/flexible endoscope reprocessor
CINAHL: the Cumulative Index to Nursing and Allied Health Literature
ESBL: extended-spectrum beta-lactamase
ERCP: endoscopic retrograde cholangiopancreatography
EWD: endoscope washer disinfector
OXA-48: oxacillinase-48
CRE: carbapenemase-resistant enterobacteriaceae
NDM 1: new delhi metallo-beta-lactamase 1
VIM-2: verona integron-encoded metallo-beta-lactamase 2
ATP: adenosine triphosphate
